# Offspring outcomes after prenatal interventions for common mental disorders: a meta-analysis

**DOI:** 10.1186/s12916-018-1192-6

**Published:** 2018-11-15

**Authors:** Marlies E. Brouwer, Alishia D. Williams, Sam E. van Grinsven, Pim Cuijpers, Mijke P. Lambregtse-van den Berg, Huibert Burger, Claudi L. H. Bockting

**Affiliations:** 1 Department of Clinical Psychology, Utrecht University, Heidelberglaan 1, 3584 CS Utrecht, the Netherlands; 2 Department of Psychiatry, Amsterdam University Medical Centres, location AMC, University of Amsterdam, Meibergdreef 9, 1105 AZ Amsterdam, the Netherlands; 3 Department of Clinical, Neuro and Developmental Psychology, Vrije Universiteit Amsterdam, Van der Boechorststraat 1, 1081 BT Amsterdam, the Netherlands; 4 Department of Psychiatry and Child and Adolescent Psychiatry/Psychology, Erasmus Medical Centre Rotterdam, P.O. Box 2060, 3000 CB Rotterdam, the Netherlands; 5 Department of General Practice, University Medical Centre Groningen, University of Groningen, Antonius Deusinglaan 1, 9713 AV Groningen, the Netherlands; 6 Faculty of Science, School of Psychology, The University of New South Wales, Sydney, NSW 2052 Australia

**Keywords:** Common mental disorders, Prenatal interventions, Offspring, Child, Pregnancy, Antidepressant, Psychotherapy, Depression, Anxiety

## Abstract

**Background:**

It is presumed that pharmacological and non-pharmacological treatment of prenatal common mental disorders can mitigate associated adverse effects in offspring, yet strong evidence for the prophylactic benefits of treatment is lacking. We therefore examined the effect of prenatal treatments for common mental disorders on offspring outcomes.

**Methods:**

For this meta-analysis, articles published up to August 31, 2017, were obtained from PubMed, PsycInfo, Embase, and Cochrane databases. Included studies needed to be randomized controlled trials (RCTs) on the effect of treatment of prenatal common mental disorders comparing an intervention to a control condition, including offspring outcome(s). Random effects models were used to calculate Hedges’ *g* in the program Comprehensive Meta-Analysis^©^ (version 3.0).

**Results:**

Sixteen randomized controlled trials among 2778 pregnant women compared offspring outcomes between prenatal interventions and control groups. There were zero pharmacological, 13 psychological, and three other interventions (homeopathy, relaxation interventions, and short psycho-education). Birth weight (mean difference 42.88 g, *g* = 0.08, 95% CI −0.06 to 0.22, *p* = 0.27, *n* = 11), Apgar scores (*g* = 0.13, 95% CI −0.28 to 0.54, *p* = 0.53, *n* = 4), and gestational age (*g* = 0.03, 95% CI −0.06 to 0.54, *p* = 0.49, *n* = 10) were not significantly affected. Other offspring outcomes could not be meta-analyzed due to the inconsistent reporting of offspring outcomes and an insufficient number of studies.

**Conclusions:**

Non-pharmacological interventions had no significant effect on birth outcomes, although this outcome should be considered with caution due to the risk of biases. No randomized controlled trial examined the effects of prenatal pharmacological treatments as compared to treatment as usual for common mental disorders on offspring outcomes. Present clinical guidelines may require more research evidence on offspring outcomes, including child development, in order to warrant the current recommendation to routinely screen and subsequently treat prenatal common mental disorders.

**Trial registration:**

PROSPERO CRD42016047190

**Electronic supplementary material:**

The online version of this article (10.1186/s12916-018-1192-6) contains supplementary material, which is available to authorized users.

## Background

Leading clinical guidelines advise to screen and treat common mental disorders and symptoms among all pregnant women [1, 2]. Common mental disorders and symptoms generally refer to mood and anxiety disorders, including depression, phobias (including extreme fear of childbirth ‘tokophobia’), generalized anxiety disorder, post-traumatic stress disorder, and obsessive-compulsive disorders [2]. Prevalence rates of mental disorders during pregnancy are estimated as high as 12.4% for mood disorders and 15.2% for anxiety disorders [3, 4]. Next to the burden of these common mental disorders for the pregnant women, these disorders may be harmful for the offspring [5]. Adverse effects on the offspring include almost 20% increased odds of low birth weight as compared to offspring from mothers without mental problems [6]. Low birth weight in turn has repeatedly been linked to negative (long term) somatic outcomes such as all-cause mortality, stunted growth, respiratory problems, and obesity [7]. In addition, low birth weight is associated with an increased risk for the development of mental problems [8]. Other adverse effects of prenatal mental disorders on the (unborn) child include a 13% increased risk of premature birth [6] and lower Apgar scores [5]. Similar to the effects of low birth weight, children from women who had a mental disorder during pregnancy have a two to three times increased risk for the development of psychopathology [9]. This includes an increased risk of symptoms of depression in (late) adolescence [10], an increased risk of anxiety in the ages 6 to 9 years old, and internalizing and externalizing (psychiatric) problems at the ages 2 to 6 [2, 11, 12]. Other risks for the offspring of pregnant women with common mental disorders include behavioral, motor, developmental, and cognitive problems such as attention-deficit hyperactivity disorder and an atypical (functional and structural) brain development [2, 9, 13, 14]. Theoretical accounts of the associations between prenatal mental health and offspring outcomes focus on a cascade of processes, such as activation of the stress-response (hypothalamic-pituitary-adrenal [HPA] axis), (epi)genetics, e.g., methylation of “stress” genes, elevated levels of intrauterine cytokines or glucocorticoids, and poor self-care during pregnancy (e.g., smoking, disturbed appetite) or poor mother-child attachment in the postpartum period due to the disabling nature of mental health problems [10, 14–18].

Effective treatments for prenatal common mental disorders are therefore of paramount importance given the recurrent and life-long course of most mental disorders and their association with (chronic) somatic conditions [19]. Although the exact mechanisms through which prenatal mental disorders exert an effect on offspring are currently unknown, the adverse outcomes themselves are clear. Current evidence-based treatments for mental disorders during pregnancy, i.e., medication and/or psychological interventions, are (implicitly) presumed to not only address mental needs of pregnant women, but also to confer prophylactic mental and physical benefits for offspring [20, 21]. As implied in the NICE guidelines, “The impact of any mental health problem may often require more urgent intervention than would usually be the case because of its potential effect on the foetus/baby (..)” [2]. The most used treatment for these disorders during pregnancy is antidepressant medication (AD; 3.7% of all pregnant women in the UK up to 6.2% in the USA [22, 23]), followed by psychological therapies including cognitive behavioral therapy (CBT) [24]. Supporting evidence for the benefits of these treatments is however limited, and the impact is typically restricted to the pregnant woman [1, 2, 24, 25]. Previous meta-analyses indicate that the evidence is restricted to psychological interventions for prenatal depression, for which CBT and interpersonal psychotherapy were shown to be most effective [24].

Given that the recommended treatments for prenatal common mental disorders might paradoxically have an adverse effect on the offspring intrauterine, it is crucial to examine the effects of prenatal maternal treatments on offspring. To our knowledge, no meta-analyses of randomized controlled trials (RCTs) have examined the effect of various treatments on common mental disorders during pregnancy on offspring [1, 2, 24, 25]. One meta-analysis indicated a positive, but small effect of prenatal preventive and acute treatments for depression on child functioning only [26]. Nonetheless, no conclusions regarding the effect of acute treatment for prenatal common mental disorders on offspring could be made, given that the majority of the studies that were included in this review included healthy pregnant women without (a history of) MDD or depressive symptoms (not acute treatment). Moreover, in the review, the authors did not assess the effects of acute treatment alone on child functioning. The last is the main aim of this meta-analysis, i.e., to examine the effect of prenatal treatments for common mental disorders on offspring outcomes. Some studies furthermore report adverse effects of prenatal antidepressants use on preterm birth, birth weight, and Apgar scores [27], persistent pulmonary hypertension (PPHN) [28, 29], development [30, 31], and cardiovascular malformations [32] in offspring. However, these reports are derived from non-randomized studies that do not permit conclusions of causality. The beneficial or possibly iatrogenic effects of psychological interventions on offspring are however less clear, despite the beneficial effects of psychotherapies for perinatal major depressive disorders as reported in previous meta-analyses [24, 25]. A review of the evidence from RCTs is timely and warranted. The primary aim of the current study was to conduct a meta-analysis to examine whether treatments for pregnant women with common mental disorders, as recommended in leading clinical guidelines (including antidepressants and psychotherapy) [1, 2], prevent adverse effects in offspring, both in terms of somatic and mental outcomes.

## Method

### Search strategy and selection criteria

This meta-analysis was conducted in accordance with the PRISMA guidelines and registered on PROSPERO [33]. A search in PubMed, PsycInfo, Embase, and the Cochrane database of randomized trials was performed on articles published from their origin through April 2016 and updated up to August 31, 2017. Five search strings were composed using standardized vocabulary (e.g., MeSH terms and text words), terms for searching title and abstract, and Boolean operators. The full search string is presented in Additional file 1. The five key strings targeted pregnancy, common mental disorders, interventions, offspring outcomes, and study design.

Included studies needed to (1) be a (cluster) randomized controlled trial; (2) treat (3) one or more prenatal common mental disorders or high levels of symptoms; (4) compare an intervention to a control condition; (5) include at least one offspring outcome; and (6) report sufficient information to calculate effect sizes (or provide this information available upon request). Included common mental disorders and symptoms were mood disorders and anxiety disorders according to the definition of DSM-IV axis I and older [34]. These included obsessive-compulsive disorders and trauma- and stress-related disorders/symptoms. Severe mental illnesses like bipolar disorder, psychosis, schizophrenia, or substance abuse were excluded due to the low prevalence rates [2]. The common mental disorder or symptoms could be assessed by self-report measures or clinical interviews, provided that the participants were primarily selected based upon the presence of mental disorders or high symptom levels. The control condition was defined as care or treatment as usual, wait-list control, or placebo medication. Language was restricted to English and Dutch due to the language proficiency of the authors. Two authors (MEB and SG) independently screened and selected the articles and assessed the risk of bias using the seven criteria as proposed by the Cochrane Handbook for Systematic Reviews of Interventions. Disagreement was solved by consultation of a third rater (CLB) and reaching consensus. The Grading of Recommendations Assessment, Development and Evaluation (GRADE) framework [35] was used to assess the overall level of certainty and strength of evidence for each of the main offspring outcomes. The evidence for each of the main offspring outcomes was potentially downgraded based upon risk of bias, inconsistency, indirectness, imprecision, number of participants, and pooled effect sizes.

### Offspring outcome measures

The primary outcomes included all offspring variables collected in the neonatal period, during infancy and early childhood. They included Apgar scores, birth weight, gestational age, and measures of cognitive, motor, and emotional development. Examples of these variables are offspring depressive and anxiety symptoms (e.g., as assessed by the strengths and difficulties questionnaire [36]), general development (e.g., Bayley scales of infant development [37]), child behavior (e.g., child behavior checklist [38]), neurodevelopmental problems (e.g., Brazelton neonatal behaviour assessment scale [39]), and biological measures (e.g., cortisol levels, height, weight). No restrictions were made on the assessment instruments (self-report, reports, observations). Offspring outcomes needed to be reported as continuous outcomes in order to be able to calculate Hedges’ *g* effect sizes. Where applicable, these outcomes were converted to the international system of units (SI), or to equal units (e.g., months to weeks, days to weeks, kilograms to grams). Data was extracted by one author (SG) using a standardized form and fully checked by a second author (MEB). Offspring measures that were reported in less than three studies were excluded from analyses.

### Data analysis

The effect sizes for all child outcomes were calculated using Hedges’ *g* and 95% confidence intervals (CI) to correct for small sample bias. Each effect size thus indicates a standardized comparison between the intervention group and control group. To calculate the pooled effect sizes, we extracted the reported mean scores, standard deviations, and number of participants for each group (intervention and control groups separately) for each offspring measurement. The software program Comprehensive Meta-Analysis^©^ (version 3.0) was used to calculate pooled effect sizes, mean differences, forest plots, heterogeneity, and funnel plots. The effect sizes were interpreted according to Cohen’s rule of thumb (small = 0.20–0.49; medium = 0.50–0.79; large = 0.80 and higher). As an indicator of heterogeneity among the effect sizes, we used the *I*^2^ statistics (0% = no heterogeneity to 75% = high heterogeneity). We calculated 95% confidence intervals [40] around *I*^2^, using the non-central *χ*^2^-based approach within the heterogi module for Stata [41]. Funnel plots were used to visually inspect for publication bias, which was statistically checked with Egger’s test of the intercept and Duval and Tweedie’s trim and fill procedure [42].

A priori, we expected substantial heterogeneity between studies and therefore used a random effects model [43] in which a pooled effect size was calculated for each offspring outcome, and one random effects model for the overall pooled effect size. Secondly, several post hoc subgroup analyses were performed, in which pooled effect sizes were calculated for the main DSM disorders or symptoms, DSM disorders or symptoms and the offspring outcomes, type of intervention (psychotherapy, supplements or medication, and other), diagnostic status (through clinical interview or self-reported symptoms), risk of bias (high, low), and whether the author indicated a significant and positive effect of the intervention on maternal main DSM disorders or symptoms (yes, no). A mixed effects model was used in the subgroup analyses, where the pooled effect sizes within subgroups were calculated with the random effects model, and the fixed effects model was used to test the difference between subgroups.

## Results

**Fig. 1:**
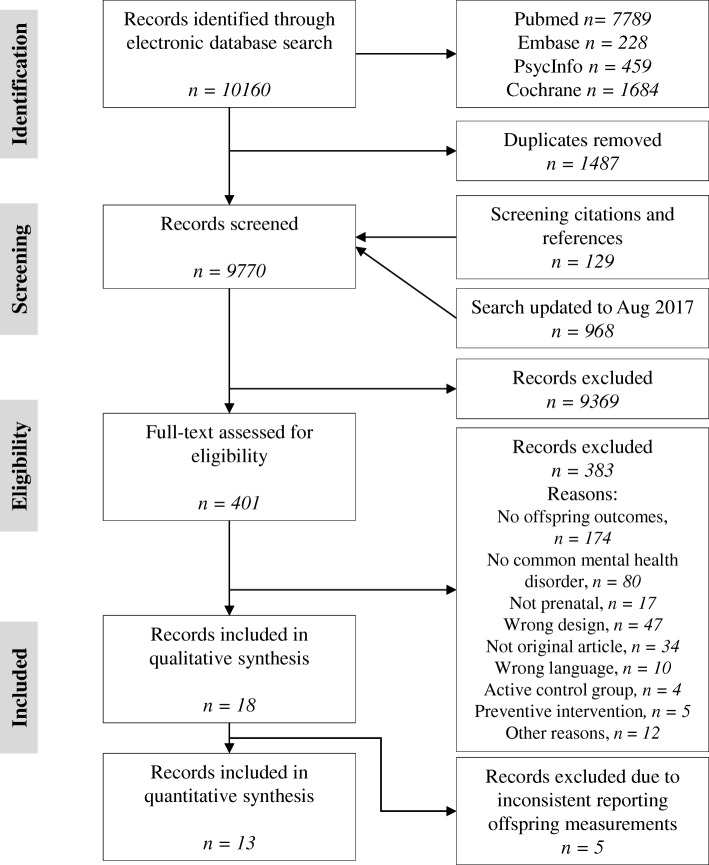
Flowchart of included studies

The search yielded 10,160 results up to April 2016. Citations and references of included articles and 207 reviews resulted in 129 additional articles. The search was updated up to August 2017, which resulted in 968 additional articles. A total of 9770 articles were screened after removal of duplicates. The systematic search resulted in 18 eligible articles (0.18%; see Fig. 1 for full details) reporting results of 16 RCTs, which are reported in Table 1. In total, 2778 pregnant women were randomized over 11 different treatment types. Treatment types included (variations of) CBT, massage therapy, psycho-education, relaxation treatments, and couples therapy. Intensity of the interventions ranged from two psycho-education phone sessions up to 16 CBT-based home visits. Details of treatment type, intensity, and the effects on maternal psychopathology are reported in Table 1. Seven studies focused on depression only [44–50], two on both depression and anxiety [51, 52], three on anxiety only [53–55], two on fear of childbirth [56, 57], one on posttraumatic stress disorder (PTSD) [58], one on stress in general [59], and one on various common mental disorders [60]. The risk of bias in the included studies was in general high, as reported in Table 1.

**Table 1:** Overview of included studies

Author, year	Study focus	Selection criteria	Conditions and timing of intervention	No. of participants	Authors conclude sign. Effect of intervention on mother?	Offspring measures	Country	Risk of bias							
								S	C	B	A	I	R	O	T
Bastani, 2006 [53]	Anxiety	STAI > 30	IG: Applied relaxation training, 7 wk. group sessions CG: TAU Prenatal	IG, 52; CG, 52; Total, 104	Yes, on anxiety symptoms	*Medical records:* BW & GA	Iran	?	?	?	?	+	–	?	H
Cappon, 2015 [54] ^x^	Anxiety	BAI ≥ 21	IG: Listening to music, 20 to 296 times individually CG: TAU Prenatal	IG, 36; CG, 37; Total, 73	Yes, on anxiety symptoms	*Questionnaire:* BW & GA. *IBQ-r* at age 2 to 3 months	USA	?	?	+	+	–	+	+	L
Chambers, 2009 [55] ^x^	Anxiety	STAI; State or trait subscale ≥ 40	IG: 6 wk. individual relaxation training CG: List of tips for reducing stress Prenatal	IG, 10; CG, 12; Total, 22	No	*Medical records:* BW & GA & Apgar scores	USA	+	?	–	+	–	+	–	H
Fenwick, 2015 [56]	Fear of childbirth	W-DEQ ≥ 66	IG: Telephone psycho-education, 2 sessions CG: TAU Prenatal	IG, 91; CG, 93; Total, 184	Yes, on flashbacks	*Medical records:* GA. Hospitalization up to 6 weeks	Australia	+	–	–	+	+	+	+	L
Field, 2009 [45]	Depression	SCID diagnosis MDD	IG: Massage therapy, 12 wk. sessions CG: TAU Prenatal	IG, 88; CG, 61; Total, 149	Yes, on depressive symptoms	*Medical records:* BW & GA. *Saliva* (cortisol) *& BNBAS* at age 2 days	USA	+	?	–	+	–	–	–	H
Karamoozian, 2015 [51]	Depression & Anxiety	EPDS > 9 & PRAQ high score	IG: 12 wk. group CBSM CG: TAU Prenatal	IG, 14; CG, 15; Total, 29	Yes, on anxiety and depressive symptoms	*Apgar scale:* Apgar scores	Iran	?	?	?	?	+	+	–	H
Madigan, 2015 [58]	PTSD in adolescents	CPTSDI PTSD diagnosis *or* AAI unresolved state of mind	IG: 12 wk. trauma focused CBT plus 12 sessions parenting course CG: Parenting course (TAU) Prenatal	IG, 12; CG, 14; Total, 26	No	*ASSP* at age 12 months	Canada	?	?	?	+	+	+	–	L
Maselko, 2015 [46] ^a*^	Depression	SCID diagnosis MDD	IG: Thinking healthy programme (CBT based), 7 wk. visits and 9 monthly visits CG: Enhanced routine care Prenatal and postpartum	IG, 289; CG, 295; Total, 584	Yes, less MDD diagnoses in IG	*SDQ, SCAS,* weight, height, and BMI at age 7 years	Pakistan	+	+	+	+	+	–	–	L
Milgrom, 2015 [50]	Depression	SCID diagnosis MDD *and* EPDS ≥ 13	IG: Beating blues before birth (CBT based), 8 sessions CG: TAU Prenatal	IG, 16; CG, 13; Total, 29	Yes, on depressive and anxiety symptoms	*Medical records:* BW & GA. *IBQ-R, ASQ-3, and ASQ-SE* at age 9 months	Australia	+	+	?	+	+	–	–	M
Netsi, 2015 [47]	Depression	CIS diagnosis MDD	IG: 12 sessions individual CBT CG: TAU Prenatal	IG, 14; CG, 11; Total, 25	Yes, on depressive symptoms	*Medical records:* BW. *ICQ and BISQ* at age 2 months	UK	+	+	–	+	–	+	–	H
Rahman, 2008 [44]*	Depression	SCID diagnosis MDD	IG: Thinking healthy programme (CBT based), 7 wk. visits and 9 monthly visits CG: Enhanced routine care Prenatal and postpartum	IG, 440; CG, 463; Total, 903	Yes, on MDD diagnoses	*Records*: Weight, height, health at ages 6 and 12 months	Pakistan	+	+	+	+	+	+	+	L
Rothberg, 1991 [59]	Stress	SRRS ≥ 39	IG: Psychosocial support during each antenatal clinic visit CG: TAU Prenatal	IG, 43; CG, 43; Total, 86	No	*Medical records & Ballard score:* BW & GA. *Height, hospitalization* at birth	South Africa	+	?	?	+	–	+	–	H
Rouhe, 2013 [57]	Fear of childbirth	W-DEQ ≥ 100	IG: 6 sessions psycho-educative group therapy CG: TAU Prenatal and postpartum	IG, 131; CG, 240; Total: 371	Yes, on fear symptoms	*Medical records:* BW & GA & Apgar scores. Arterial Ph. at birth	Finland	+	+	–	–	–	+	–	H
Urizar, 2011 [48]	Depression	CES-D ≥ 16 *or* past history of MDD	IG: 12 wk. group CBSM CG: TAU Prenatal	IG, 24; CG, 29; Total, 53	No	*Saliva* (cortisol) at ages 6 and 18 months	USA	?	?	?	?	+	+	–	H
Verbeek, 2016[52]^x^	Depression & Anxiety	EPDS ≥ 12 *or* STAI ≥ 42	IG: 10 to 12 sessions individual CBT CG: TAU Prenatal and postpartum	IG, 121; CG, 120; Total, 241	No	*Medical records:* BW & GA & Apgar scores	NL	+	+	–	+	+	+	+	L
Verbeek, 2016b [52] ^b x^	Depression & Anxiety	EPDS ≥ 12 *or* STAI ≥ 42	IG: 10 to 12 sessions individual CBT CG: TAU Prenatal and postpartum	IG, 97; CG, 99; Total, 196	No	*CBCL and BSID* at age 18 months	NL	+	+	–	+	+	+	+	L
Vilhena, 2017 [60]	Common mental disorder in obese women	SRQ-20 ≥ 8	IG: Homeopathy, 2 times a day, 4 days a week CG: Placebo Prenatal	IG, 62; CG, 72; Total, 134	No	*Records:* GA, BW, Apgar of 10 at 5 min after birth	Brazil	+	+	+	?	?	+	–	L
Zhao, 2017 [49]	Depression	EPDS ≥ 9 or PDSS ≥ 20	IG: 6 sessions couple-separated psycho-educational program for first time parents CG: TAU Prenatal	IG, 175; CG, 174; Total, 349	Yes, on depressive status	*Medical records:* GA, BW	China	+	?	?	?	+	+	?	L

All presented studies were randomized controlled trials (RCT), except for *cluster RCT. ^x^Doctoral dissertation. ^a^Follow-up for Rahman 2008. ^b^Follow-up for Verbeek 2016. *IG* intervention group, *CG* control group, *TAU* treatment as usual, *wk* weekly, *MDD* major depressive disorder, *CBSM* cognitive-behavioral stress management, *CBT* cognitive behavioral therapy, *BW* birth weight, *GA* gestational age, *USA* United States of America, *UK* United Kingdom, *NL* the Netherlands, *BAI* Beck anxiety inventory, *STAI* state trait anxiety inventory, *W-DEQ* Wijma delivery expectancy/experience questionnaire, *SCID* structured clinical interview for DSM disorders, *EPDS* Edinburgh postnatal depression scale, *PRAQ* pregnancy-related anxiety questionnaire, *CPTSDI* children’s PTSD inventory, *PTSD* posttraumatic stress disorder, *AAI* adult attachment interview, *MDD* major depressive disorder, *CIS* clinical interview schedule, *SRRS* social readjustment rating scale, *CES-D* Centre for Epidemiological Studies depression Scale, *SRQ-20* self-report questionnaire, *IBQ-R* infant behaviour questionnaire revised, *ASQ-3* ages and stages questionnaire, *ASQ-SE* ages and stages questionnaire social emotional, *SDQ* strengths and difficulties questionnaire, *SCAS* Spence children’s anxiety scale, *ASSP* Ainsworth strange situation procedure, *BNBAS* Brazelton neonatal behaviour assessment scale, *ICQ* infant characteristic questionnaire, *BISQ* brief infant sleep questionnaire, *BSID* Bayley scales of infant development, *CBCL* child behaviour checklist. Risk of bias criteria are represented as *S* = sequence generation, C = allocation concealment, B = blinding of participants and personnel, A = blinding of outcome assessors, I = incomplete outcome data, R = selective reporting, O = other sources of bias, and are coded as + = low risk of bias (0 points), − = high risk of bias (2 points),? = unclear risk of bias (1 point). T = total risk of bias, in which H = high risk of bias, L = low risk of bias. Total risk of bias is scored as < 6 = low risk, > 6 high risk

**Fig. 2:**
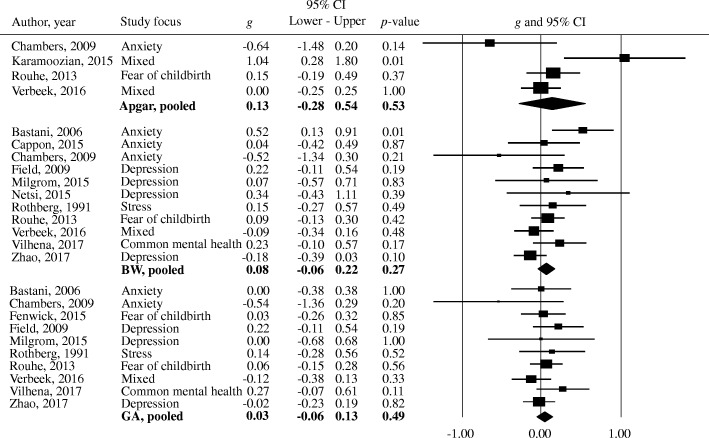
Forest plots for Apgar scores, birth weight, and gestational age. BW = birth weight, GA = gestational age, *g* = Hedges’ *g*, mixed = combination of multiple common mental disorders and/or symptoms

**Fig. 3:**
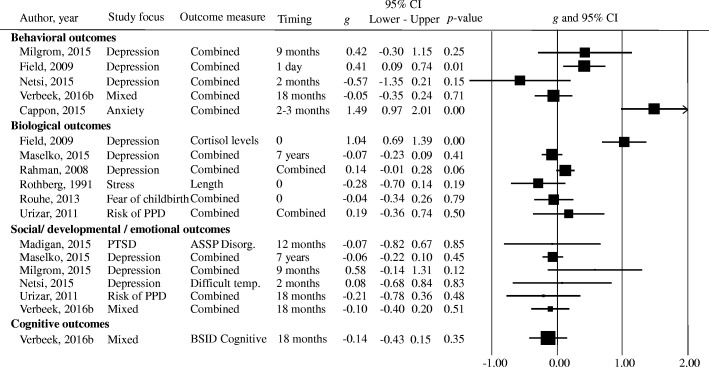
Forest plots of excluded offspring outcomes*. g* = Hedges’ *g*, mixed = combination of multiple common mental disorders and/or symptoms, combined = combination of multiple offspring outcomes and/or timing of offspring outcomes, PPD = peripartum depression, ASSP Disorg = Ainsworth stranger situation disorganized attachment measure, BSID Cognitive = Bayley scales of infant development-cognitive subscale, difficult temp = difficult temperament

The included studies reported 28 different offspring outcome measures. Most outcomes were reported in a single study only. Analyses were restricted to the outcomes that were reported in at least three studies, which resulted in three eligible outcomes: birth weight, Apgar (1, 5, and 10 min combined), and gestational age (Fig. 2). For five studies, it was therefore not possible to aggregate any of the reported offspring outcomes in the meta-analysis, both in terms of target outcome (e.g., cognitive vs. emotional development) and measurement instrument. Effect sizes of the removed outcomes for each study are reported in Fig. 3. The mean values for each included study, and the overall highest and lowest values on the three offspring outcomes are displayed in Table 2.

Since there were no RCTs of pharmacological interventions during pregnancy reporting offspring outcomes, data from 11 non-pharmacological RCTs (reported in 13 articles) were analyzed. First, pooled effect sizes for each selected outcome were calculated. Outcomes of the meta-analysis are reported in Table 3 and displayed in Fig. 2. Birth weight was not significantly affected by interventions as compared to control conditions, corresponding to a mean difference of 42.88 g (95% CI − 33.06 to 118.83, *N* = 11, *n* = 1583, *I*^2^ = 39% [45, 47, 49, 50, 52–55, 57, 59, 60]) between the intervention and control groups. Gestational age and Apgar scores did not significantly improve or worsen by prenatal interventions (gestational age mean difference = 0.08 weeks [95% CI −0.09 to 0.24], *N* = 10, *n* = 1669, *I*^2^ = 0% [49, 50, 52, 53, 55–57, 59, 60]; Apgar mean difference = 0.21 [95% CI −0.28 to 0.71], *N* = 4, *n* = 663, *I*^2^ = 68% [51, 52, 55, 57]). The pooled effect size indicated an overall non-significant effect on Apgar scores, birth weight, and gestational age together. Since these three offspring outcomes are heterogeneous in interpretation, this overall result is not further discussed. Heterogeneity across the outcomes was low, *I*^2^ = 23% (95% CI 0–60%). There was some indication of publication bias, as indicated by Duval and Tweedie’s trim and fill procedure (studies trimmed = 2, adjusted *g* = 0.05, 95% CI −0.05 to 0.15). Visual inspection of the funnel plot and the Egger’s test (*p* = 0.16) did not indicate publication bias. The evidence for each of the three pooled effect sizes was downgraded using the GRADE assessment to very low certainty of evidence. For each of the outcomes, there was a serious risk of bias, consistency in measures, serious indirectness, and serious imprecision. Birth weight and gestational age were rated as important outcomes, and Apgar scores were rated as not important.

**Table 2:** Study offspring outcomes

Author, year	Mean birth weight (Sd.) in grams	Mean Apgar score (Sd.)	Mean gestational age (Sd.) in weeks
Bastani, 2006 [53]	IG, 3168 (420) CG, 2883 (640)	n/a	IG, 38 (5.9) CG, 38 (4.4)
Cappon, 2015 [54]	IG, 3366 (523) CG, 3344 (773)	n/a	n/a
Chambers, 2009 [55]	IG, 3232 (695) CG, 3562 (529)	IG, Apgar 1 min: 7.8 (1.3) CG, Apgar 1 min: 8 (1.1) IG, Apgar 5 min: 8.5 (0.7) CG, Apgar 5 min: 9.1 (0.3)	IG, 38.4 (2.5) CG, 39.5 (1.4)
Fenwick, 2015 [56]	n/a	n/a	IG, 39.2 (1.6) CG, 39.1 (2.0)
Field, 2009 [45]	IG, 3318 (360) CG, 3226 (492)	n/a	IG, 39 (1.2) CG, 38.7 (1.6)
Karamoozian, 2015 [51]	n/a	IG, Apgar 1 min: 8.9 (0.3) CG, Apgar 1 min: 8.1 (0.8) IG, Apgar 5 min: 9.7 (0.5) CG, Apgar 5 min: 9.3 (0.7)	n/a
Milgrom, 2015 [50]	IG, 3626 (786) CG, 3575 (595)	n/a	IG, 40 (1) CG, 40 (2)
Netsi, 2015 [47]	IG, 3423 (503) CG, 3180 (871)	n/a	n/a
Rothberg, 1991 [59]	IG, 3214 (649) CG, 3113 (690)	n/a	IG, 38.7 (3) CG, 38.3 (2.7)
Rouhe, 2013 [57]	IG, 3532 (550) CG, 3486 (518)	IG, Apgar 1 min: 8.7 (1.1) CG, Apgar 1 min: 8.5 (1.4) IG, Apgar 5 min: 9.3 (1.0) CG, Apgar 5 min: 9.1 (1.2) IG, Apgar 10 min: 9.7 (0.8) CG, Apgar 10 min: 9.5 (0.9)	IG, 39.7 (1.5) CG, 39.6 (1.6)
Verbeek, 2016; Verbeek, 2016b [52]	IG, 3419 (651) CG, 3474 (561)	IG, Apgar 1 min: 8.6 (n/a) CG, Apgar 1 min: 8.6 (n/a) IG, Apgar 5 min: 9.5 (n/a) CG, Apgar 5 min: 9.5 (n/a) IG, Apgar 10 min: 9.8 (n/a) CG, Apgar 10 min: 9.8 (n/a)	IG, 38.9 (2.3) CG, 39.2 (1.8)
Vilhena, 2017 [60]	IG, 3390 (440) CG, 3270 (560)	n/a	IG, 39.4 (1.2) CG, 38.9 (1.9)
Zhao, 2017 [49]	IG, 3256 (522) CG, 3349 (529)	n/a	n/a
Combined range per offspring outcome	Lowest value, 2883 Highest value, 3626	Lowest value, 7.8 Highest value, 9.8	Lowest value, 38 Highest value, 40

*IG* intervention group, *CG* control group

**Table 3:** Meta-analyses and subgroup analyses results

		*N*	No. of participants	*g*	95% CI	*p*	*I* ^2^	95% CI	*p* ^a^
Effect of prenatal interventions on offspring outcomes (BW, GA, Apgar)	All studies [45, 47, 49–57, 59, 60]	13	1796	0.09	− 0.03 to 0.21	0.14	23	0–60	
Effect of prenatal interventions for each offspring outcome	BW [45, 47, 49, 50, 52–55, 57, 59]	11	1583	0.08	− 0.06 to 0.22	0.27	39	0–70	
	GA [49, 50, 52, 53, 55–57, 59, 60]	10	1669	0.03	− 0.06 to 0.13	0.49	0	0–53	
	Apgar [51, 52, 55, 57]	4	663	0.13	− 0.28 to 0.54	0.53	68	0–87	
Subgroup analyses									
Type of diagnosis*	Anxiety [52–57]	6	852	− 0.01	− 0.22 to 0.20	0.89	40	0–75	0.41
	BW [52–55, 57]	5	668	0	− 0.33 to 0.32	1	70	22–88	
	GA [52–56]	5	779	− 0.06	− 0.24 to 0.12	0.54	27	0–71	
	Depression [45, 47–50]	5	575	0.02	− 0.15 to 0.18	0.86	0	0–79	
	BW [45, 47–50]	5	575	0.01	− 0.20 to 0.23	0.90	22	0–68	
	GA [45, 48–50]	4	550	0.05	− 0.12 to 0.21	0.58	0	0–85	
	Other [51, 52, 59, 60]	4	283	0.22	− 0.90 to 0.54	0.17	62	0–85	
	BW [52, 59, 60]	3	254	0.06	− 0.15 to 0.27	0.57	22	0–97	
	GA [52, 59, 60]	3	254	0.07	− 0.19 to 0.33	0.60	45	0–84	
Risk of bias	High [45, 47, 51, 53, 55, 57, 59]	7	786	0.21	− 0.00 to 0.42	*0.05*	31	0–70	0.09
	Low [49, 50, 52, 54, 56, 60]	6	1010	− 0.00	− 0.13 to 0.12	0.97	0	0–61	
Diagnosis through clinical interview or self-report	Clinical interview [45, 47, 50]	3	203	0.20	− 0.07 to 0.47	0.15	0	0–90	0.43
	Self-report [49, 51–60]	10	1593	0.08	− 0.06 to 0.22	0.28	36	0–70	
Type of intervention	Psychotherapy [47, 49–52, 57, 59]	7	1130	0.08	− 0.10 to 0.27	0.40	39	0–75	0.67
	Other [45, 53–56, 60]	6	666	0.13	− 0.02 to 0.28	0.09	0	0–74	
Report of sign. Effect of intervention on mother?	No [52, 55, 59, 60]	4	483	0.05	− 0.18 to 0.28	0.68	31	0–75	0.64
	Yes [45, 47, 49–51, 53, 54, 56, 57]	9	1313	0.11	− 0.04 to 0.26	0.14	29	0–67	

^a^The *p* value indicates whether the subgroups differ from each other. *g* = Hedges’ *g*, 95% CI = confidence interval 95% of Hedges’ *g*. *In this subgroup analysis, the Verbeek 2016 study subgroups depression, anxiety, and combined (other) were analyzed separately. *BW* birth weight, *GA* gestational age. Superscript numbers refer to included studies in each analysis

Table 3 displays several subgroup analyses. Studies targeting depression and anxiety, or with a focus on other disorders, did not show a significant pooled effect size, hence did not improve offspring outcomes based on the three selected outcomes. According to the mixed effects analysis, the subgroups did not differ significantly. When investigating the separate offspring outcomes for each disorder (depression, anxiety, and other), it appeared that there was no significant effect of intervention on offspring outcomes within each disorder. The overall effect size in the risk of bias subgroup analysis was not significant. Although studies with a high risk of bias were significantly related to positive birth outcomes (*g* = 0.21, 95% CI 0.00 to 0.42, *p* = 0.05, *N* = 7, *n* = 786, *I*^2^ = 31% [45, 47, 51, 53, 55, 57, 59]), the mixed effects analysis indicated no significant difference between high and low risk of bias studies (*p* = 0.09). The method through which the (possible) diagnosis was established, i.e., through clinical interview or self-report, whether the authors reported that the intervention had a significant effect on treating maternal common mental disorder (symptoms) compared to the control group, and the type of intervention (psychotherapy versus other types), did not significantly affect offspring outcomes, nor did the mixed effects analyses show differences between the subgroups (Table 3).

## Discussion

Leading international clinical guidelines [1, 2] recommend screening and treatment of pregnant women for mental disorders and symptoms, with the clinical view to additionally prevent adverse effects on the offspring. The systematic search identified 16 non-pharmacological RCTs, including 14 psychological interventions that reported offspring outcomes. The results from the current meta-analysis indicate that non-pharmacological interventions do not have a significant effect on improving birth outcomes, and their effects could not be meta-analyzed for (developmental) offspring outcomes. Therefore, based on the current evidence, there is no strong indication of prophylactic effects. Although no statistically significant result was obtained for birth weight, all effect sizes within the 95% confidence interval around the pooled effect estimate were small, indicating that the true effect of prenatal interventions on birth weight is most likely small. Furthermore, the quality of most studies was low and the studies were relatively heterogeneous. An explanation for the non-significant effect size may be a ceiling effect since offspring in the control and intervention groups had a birth weight within the normal range. The birth weight in the trials ranged from 2883 to 3626 g, indicating there was no low birth weight according to the worldwide standards for low birth weight, that is less than 2500 g [61].

Secondly, the meta-analysis did not show that the somatic outcomes indexed by Apgar scores and gestational age were significantly associated with the non-pharmacological interventions. As for birth weight, the confidence intervals around the pooled effect estimates for Apgar scores and gestational age indicate that a true effect is most likely small. It was furthermore not possible to investigate the impact of prenatal interventions on preventing preterm birth, since the included studies had different definitions of preterm birth (different gestational age), or did not report enough information to calculate effect sizes. Additionally, five out of 16 studies reported different measures of (long term) offspring outcomes. It was therefore not possible to investigate the effects of interventions on non-somatic outcomes, such as psychopathology or developmental problems. Although previous research and clinical guidelines [1, 2] report on harmful effects of prenatal mental disorders on offspring, including an increased risk of low birth weight [6], the current meta-analysis could not support the notion that treatment can mitigate this effect. Furthermore, the current results did not indicate that pregnant women in the care as usual or placebo groups had worse or better offspring outcomes. This is to some extent in contrast to a recent meta-analysis, in which it was concluded that prenatal preventive and acute interventions for MDD (symptoms) improved overall child functioning. This effect was however primarily based upon preventive interventions and offspring behaviour [26].

The current meta-analysis found zero RCTs on the effects of antenatal treatment of common mental disorders with medication (i.e., antidepressants) on offspring. This is noteworthy given the fact that antidepressant medication is one of the most used treatments during pregnancy [22]. Furthermore, previous reviews of non-randomized trials indicate an association between prenatal antidepressant use and adverse offspring outcomes, including lower birth weight, preterm birth, lower Apgar scores [27, 62], and cardiovascular malformations [32]. Correspondingly, untreated maternal mental disorders has been associated with similar adverse effects, which in turn are linked to other subsequent offspring problems [7]. Other studies suggest that the effects of antidepressants on offspring might be minimal [63–65]. Due to the nature of the studies (non-randomized cohort trials), there is insufficient evidence of the possible effects of prenatal antidepressant usage. It is therefore not clear what the net effects of antidepressants are for offspring. RCTs and comparative treatment trials are needed to disentangle whether these offspring outcomes are related to antidepressant use or are predominantly the result of the mental disorders of the mother. To estimate the (enduring) relative effects of pharmacological and non-pharmacological interventions on offspring, a RCT comparing the two intervention types may provide more information on the prophylactic effects on offspring. Such design would be more ethical since the pregnant woman receives treatment according to clinical recommendations. Moreover, it would provide more information on the effects of antidepressants.

Collectively, the findings of the meta-analysis indicate that there is insufficient data to support the beneficial effects of prenatal treatments on offspring and that more research on the effects of prenatal treatments on offspring is needed. These results of the meta-analysis must be interpreted in the context of some limitations. In general, the included trials had a high risk of bias and reported different offspring outcomes, and the GRADE assessment indicated very low certainty of evidence, thereby limiting the results. Overall, there is some indication that non-pharmacological interventions may have a positive influence on offspring birth weight; however, the effect was not significant and could be overestimated due to the small samples sizes and high risk of bias. The small sample size of pregnant women, and the small amount of included studies, increases the risk of false-positive and false-negative birth outcomes. Furthermore, other birth outcomes including preterm birth, low birthweight for gestational age, or child development could not be analyzed due to the inconsistent reporting and lack of studies. There is no conclusive evidence that interventions aimed to target prenatal mental disorders are beneficial or iatrogenic for offspring, especially with regard to long-term and psychological impact.

For health care professionals, there is little evidence that prenatal non-pharmacological interventions are beneficial for the offspring with respect to birth weight, gestational age, and Apgar scores. There is not enough support that commonly used treatments for prenatal mental disorders are beneficial to offspring and hence do not provide a scientific foundation to support recommendations of specific treatment options with respect to the benefit for the child. More systematic research with long-term follow-up of the offspring is consequently needed to support (inter)national guidelines for prenatal mental disorders. As a first step, research on prenatal interventions may register birth outcomes from birth reports of (former) participants in RCTs. RCTs focusing on pharmacological interventions as compared to psychotherapy during pregnancy are needed, even though this may be challenging due to ethical considerations and preferences of pregnant women and their health care providers.

## Conclusions

The results from the current meta-analysis indicate no significant effects of non-pharmacological interventions on improving birth outcomes. No firm conclusion of prophylactic effects can be drawn due to the reported limitations. Despite the recommendation of leading international clinical guidelines [1, 2] to routinely screen pregnant women for common mental disorders and symptoms, and subsequently treat the mother to reduce perinatal symptomatology and prevent adverse effects on the offspring, there is insufficient data to support the clinical recommendation regarding the safety of prenatal treatments for the offspring. Prior research implies that prenatal interventions improve maternal psychopathology [24, 26], yet more research is warranted to draw stronger conclusions on the impact of prenatal interventions on offspring, especially regarding child development. Potential adverse effects on offspring cannot be ruled out, thereby underscoring the urgent need for properly controlled trials to best inform care approaches for mothers and their offspring.

## Additional file

Additional file 1: Search terms (DOC 46 kb)

## Abbreviations

AD: Antidepressant medication
CBT: Cognitive behavioral therapy
CI: Confidence intervals
PPHN: Persistent pulmonary hypertension
PTSD: Posttraumatic stress disorder
RCT: Randomized controlled trial

## References

[1] O’Connor E, Rossom RC, Henninger M, Groom HC, Burda BU (2016). Primary care screening for and treatment of depression in pregnant and postpartum women. JAMA.

[2] National Institute for Health and Clinical Excellence. Antenatal and postnatal mental health: clinical management and service guidance: Updated edition. NICE Clinical Guideline 192. London: NICE; 2014. Available from: https://www.nice.org.uk/guidance/cg192/evidence/full-guideline-pdf-4840896925. 31841290

[3] Dennis C-L, Falah-Hassani K, Shiri R (2017). Prevalence of antenatal and postnatal anxiety: systematic review and meta-analysis. Br J Psychiatry.

[4] Bennett HA, Einarson A, Taddio A, Koren G, Einarson TRT (2004). Prevalence of depression during pregnancy: systematic review. Obstet Gynecol.

[5] Alder J, Fink N, Bitzer J, Hösli I, Holzgreve W (2007). Depression and anxiety during pregnancy: a risk factor for obstetric, fetal and neonatal outcome? A critical review of the literature. J Matern Neonatal Med.

[6] Grote NK, Bridge JA, Gavin AR, Melville JL, Iyengar S, Katon WJ (2010). A meta-analysis of depression during pregnancy and the risk of preterm birth, low birth weight, and intrauterine growth restriction. Arch Gen Psychiatry.

[7] Belbasis L, Savvidou MD, Kanu C, Evangelou E, Tzoulaki I (2016). Birth weight in relation to health and disease in later life: an umbrella review of systematic reviews and meta-analyses. BMC Med.

[8] Loret de Mola C, Araujo de Franca GV, de Quevedo LA, Horta BL (2014). Low birth weight, preterm birth and small for gestational age association with adult depression: systematic review and meta-analysis. Br J Psychiatry.

[9] O’Donnell KJ, Glover V, Barker ED, O’Connor TG (2014). The persisting effect of maternal mood in pregnancy on childhood psychopathology. Dev Psychopathol.

[10] Stein A, Pearson RM, Goodman SH, Rapa E, Rahman A, McCallum M (2014). Effects of perinatal mental disorders on the fetus and child. Lancet.

[11] Newman L, Judd F, Olsson CA, Castle D, Bousman C, Sheehan P (2016). Early origins of mental disorder - risk factors in the perinatal and infant period. BMC Psychiatry.

[12] Lahti M, Savolainen K, Tuovinen S, Pesonen A-K, Lahti J, Heinonen K (2017). Maternal depressive symptoms during and after pregnancy and psychiatric problems in children. J Am Acad Child Adolesc Psychiatry.

[13] van den Bergh BRH, Dahnke R, Mennes M (2018). Prenatal stress and the developing brain: risks for neurodevelopmental disorders. Dev Psychopathol.

[14] van den Bergh BRH, van den Heuvel MI, Lahti M, Braeken M, de Rooij SR, Entringer S, et al. Prenatal developmental origins of behavior and mental health: the influence of maternal stress in pregnancy. Neurosci Biobehav Rev. 2017. [Epub ahead of print] 10.1016/j.neubiorev.2017.07.003 28757456

[15] Talge NM, Neal C, Glover V (2007). Antenatal maternal stress and long-term effects on child neurodevelopment: how and why?. J Child Psychol Psychiatry Allied Discip.

[16] Gluckman PD, Hanson MA, Cooper C, Thornburg KL (2008). Effect of in utero and early-life conditions on adult health and disease. N Engl J Med.

[17] Pearson RM, Evans J, Kounali D, Lewis G, Heron J, Ramchandani PG (2013). Maternal depression during pregnancy and the postnatal period: risks and possible mechanisms for offspring depression at 18 years. JAMA Psychiatry.

[18] Barker DJ (1990). The fetal and infant origins of adult disease. BMJ.

[19] Bockting CL, Hollon SD, Jarrett RB, Kuyken W, Dobson K (2015). A lifetime approach to major depressive disorder: the contributions of psychological interventions in preventing relapse and recurrence. Clin Psychol Rev.

[20] Rahman A, Fisher J, Bower P, Luchters S, Tran T, Yasamy MT (2013). Interventions for common perinatal mental disorders in women in low- and middle-income countries: a systematic review and meta-analysis. Bull World Health Organ.

[21] Lewis AJ, Galbally M, Gannon T, Symeonides C (2014). Early life programming as a target for prevention of child and adolescent mental disorders. BMC Med.

[22] Charlton RA, Jordan S, Pierini A, Garne E, Neville AJ, Hansen AV (2015). Selective serotonin reuptake inhibitor prescribing before, during and after pregnancy: a population-based study in six European regions. BJOG.

[23] Ko JY, Farr SL, Dietz PM, Robbins CL (2012). Depression and treatment among U.S. pregnant and nonpregnant women of reproductive age, 2005–2009. J Womens Heal.

[24] van Ravesteyn LM, Lambregtse-van den Berg MP, WJG H, Kamperman AM (2017). Interventions to treat mental disorders during pregnancy: a systematic review and multiple treatment meta-analysis. PLoS One.

[25] Cuijpers P, Weitz E, Karyotaki E, Garber J, Andersson G (2014). The effects of psychological treatment of maternal depression on children and parental functioning: a meta-analysis. Eur Child Adolesc Psychiatry.

[26] Goodman SH, Cullum KA, Dimidjian S, River LM, Kim CY (2018). Opening windows of opportunities: evidence for interventions to prevent or treat depression in pregnant women being associated with changes in offspring’s developmental trajectories of psychopathology risk. Dev Psychopathol.

[27] Ross LE, Grigoriadis S, Mamisashvili L, Vonderporten EH, Roerecke M, Rehm J (2013). Selected pregnancy and delivery outcomes after exposure to antidepressant medication: a systematic review and meta-analysis. JAMA Psychiatry.

[28] Huybrechts KF, Bateman BT, Palmsten K, Desai RJ, Patorno E, Gopalakrishnan C (2015). Antidepressant use late in pregnancy and risk of persistent pulmonary hypertension of the newborn. JAMA.

[29] Grigoriadis S, Vonderporten EH, Mamisashvili L, Tomlinson G, Dennis C-L, Koren G (2014). Prenatal exposure to antidepressants and persistent pulmonary hypertension of the newborn: systematic review and meta-analysis. BMJ.

[30] Rai D, Lee BK, Dalman C, Newschaffer C, Lewis G, Magnusson C (2017). Antidepressants during pregnancy and autism in offspring: population based cohort study. BMJ.

[31] Man KKC, Chan EW, Ip P, Coghill D, Simonoff E, Chan PKL (2017). Prenatal antidepressant use and risk of attention-deficit/hyperactivity disorder in offspring: population based cohort study. BMJ.

[32] Grigoriadis S, VonderPorten EH, Mamisashvili L, Roerecke M, Rehm J, Dennis C-L (2013). Antidepressant exposure during pregnancy and congenital malformations: is there an association?. J Clin Psychiatry.

[33] van Grinsven S, Brouwer ME, CLH B. Offspring outcomes of prenatal treatment of maternal psychopathology. PROSPERO. 2016; http://www.crd.york.ac.uk/PROSPERO/display_record.asp?ID=CRD42016047190.

[34] American Psychiatric Association. Diagnostic and statistical manual of mental disorders (4th ed., text rev.). Washington, DC, US: Author; 2000.

[35] Guyatt G, Oxman AD, Akl EA, Kunz R, Vist G, Brozek J (2011). GRADE guidelines: 1. Introduction—GRADE evidence profiles and summary of findings tables. J Clin Epidemiol.

[36] Goodman R (1997). The strengths and difficulties questionnaire: a research note. J Child Psychol Psychiatry.

[37] Bayley N (2006). Bayley scales of infant and toddler development.

[38] Rescorla LA (2005). Assessment of young children using the Achenbach System of Empirically Based Assessment (ASEBA). Ment Retard Dev Disabil Res Rev.

[39] Brazelton TB (1978). Introduction. Monogr Soc Res Child Dev.

[40] Ioannidis JPA, Patsopoulos NA, Evangelou E (2007). Uncertainty in heterogeneity estimates in meta-analyses. Br Med J.

[41] Orsini N, Bottai M, Higgins J, Buchan I. HETEROGI: Stata module to quantify heterogeneity in a meta-analysis. Statistical Software Components S449201, Boston College Department of Economics, revised 25 Jan 2006.

[42] Duval S, Tweedie R (2000). Trim and fill: a simple funnel-plot-based method of testing and adjusting for publication bias in meta-analysis. Biometrics.

[43] Borenstein M, Hedges LV, JPT H, Rothstein HR (2009). An introduction to meta-analysis.

[44] Rahman A, Malik A, Sikander S, Roberts C, Creed F (2008). Cognitive behaviour therapy-based intervention by community health workers for mothers with depression and their infants in rural Pakistan: a cluster-randomised controlled trial. Lancet.

[45] Field T, Diego M, Hernandez-Reif M, Deeds O, Figueiredo B (2009). Pregnancy massage reduces prematurity, low birthweight and postpartum depression. Infant Behav Dev.

[46] Maselko J, Sikander S, Bhalotra S, Bangash O, Ganga N, Mukherjee S (2015). Effect of an early perinatal depression intervention on long-term child development outcomes: follow-up of the Thinking Healthy Programme randomised controlled trial. Lancet Psychiatry.

[47] Netsi E, Evans J, Wulff K, O’Mahen H, Ramchandani PG (2015). Infant outcomes following treatment of antenatal depression: findings from a pilot randomized controlled trial. J Affect Disord.

[48] Urizar GG, Muñoz RF (2011). Impact of a prenatal cognitive-behavioral stress management intervention on salivary cortisol levels in low-income mothers and their infants. Psychoneuroendocrinology.

[49] Zhao Y, Munro-Kramer ML, Shi S, Wang J, Luo J (2017). A randomized controlled trial: effects of a prenatal depression intervention on perinatal outcomes among Chinese high-risk pregnant women with medically defined complications. Arch Womens Ment Health.

[50] Milgrom J, Holt C, Holt CJ, Ross J, Ericksen J, Gemmill AW (2015). Feasibility study and pilot randomised trial of an antenatal depression treatment with infant follow-up. Arch Womens Ment Health.

[51] Karamoozian M, Askarizadeh G (2015). Impact of prenatal cognitive-behavioral stress management intervention on maternal anxiety and depression and newborns’ Apgar scores. Iran J Neonatol.

[52] Verbeek T (2016). Pregnancy and psychopathology.

[53] Bastani F, Hidarnia A, Montgomery KS, Aguilar-Vafaei ME, Kazemnejad A (2006). Does relaxation education in anxious primigravid Iranian women influence adverse pregnancy outcomes? A randomized controlled trial. J Perinat Neonatal Nurs.

[54] Cappon R (2015). Anxious origins: attenuating maternal and fetal anxiety with acoustically modified music.

[55] Chambers AS (2009). Relaxation during pregnancy to reduce stress and anxiety and their associated complications.

[56] Fenwick J, Toohill J, Gamble J, Creedy DK, Buist A, Turkstra E (2015). Effects of a midwife psycho-education intervention to reduce childbirth fear on women’s birth outcomes and postpartum psychological wellbeing. BMC Pregnancy Childbirth.

[57] Rouhe H, Salmela-Aro K, Toivanen R, Tokola M, Halmesmäki E, Saisto T (2013). Obstetric outcome after intervention for severe fear of childbirth in nulliparous women - randomised trial. BJOG An Int J Obstet Gynaecol.

[58] Madigan S, Vaillancourt K, McKibbon A, Benoit D (2015). Trauma and traumatic loss in pregnant adolescents: the impact of trauma-focused cognitive behavior therapy on maternal unresolved states of mind and posttraumatic stress disorder. Attach Hum Dev.

[59] Rothberg AD, Lits B (1991). Psychosocial support for maternal stress during pregnancy: effect on birth weight. Am J Obstet Gynecol.

[60] de Vilhena EC, de Castilho EA (2016). Homeopathic treatment of overweight and obesity in pregnant women with mental disorders: a double-blind, controlled clinical trial. Altern Ther Health Med.

[61] United Nations Children’s Fund and World Health Organization (2004). Low birthweight: country, regional and global estimates.

[62] Grigoriadis S, VonderPorten EH, Mamisashvili L, Eady A, Tomlinson G, Dennis C-L (2013). The effect of prenatal antidepressant exposure on neonatal adaptation. J Clin Psychiatry.

[63] Huybrechts KF, Palmsten K, Avorn J, Cohen LS, Holmes LB, Franklin JM (2014). Antidepressant use in pregnancy and the risk of cardiac defects. N Engl J Med.

[64] Brown HK, Ray JG, Wilton AS, Lunsky Y, Gomes T, Vigod SN (2017). Association between serotonergic antidepressant use during pregnancy and autism spectrum disorder in children. JAMA.

[65] Sujan AC, Rickert ME, Oberg AS, Quinn PD, Hernandez-Diaz S, Almqvist C (2017). Associations of maternal antidepressant use during the first trimester of pregnancy with preterm birth, small for gestational age, autism spectrum disorder, and attention-deficit/hyperactivity disorder in offspring. JAMA.

