# Supplementary material for: Offspring outcomes after prenatal interventions for common mental disorders: a meta-analysis
Source: BMC Med. 2018 Nov 15;16:208. doi: 10.1186/s12916-018-1192-6 (PMC6237028; doi:10.1186/s12916-018-1192-6)
Supplement: Supplementary file 1 — Search terms (DOC 46 kb) [file 12916_2018_1192_MOESM1_ESM.doc]

**Additional file 1. Search terms**

**Database: Pubmed**

((((((((((((((((((((((Pregnancy[MeSH Terms]) OR peripartum period[MeSH Terms]) OR pregnancy[Title/Abstract]) OR pregnancies[Title/Abstract]) OR

pregnant[Title/Abstract]) OR perinatal[Title/Abstract]) OR

peripartum[Title/Abstract]) OR prenatal[Title/Abstract]) OR

prepartum[Title/Abstract]) OR antenatal[Title/Abstract]) OR

antepartum[Title/Abstract]) OR postnatal[Title/Abstract]) OR

postpartum[Title/Abstract]) OR post-partum[Title/Abstract]) OR

trimester[Title/Abstract]))))

*And*

((((((((((((((((((((((((("Depression"[Mesh]) OR "Depressive Disorder"[Mesh]) OR

"Mood Disorders"[Mesh]) OR "Anxiety Disorders"[Mesh]) OR "Anxiety"[Majr]) OR

("Trauma and Stressor Related Disorders"[Mesh])) OR depress*[Title/Abstract]) OR

mood[Title/Abstract]) OR dysthym*[Title/Abstract]) OR "seasonal

affective"[Title/Abstract]) OR anxiety[Title/Abstract]) OR anxious[Title/Abstract])

OR agoraphobi*[Title/Abstract]) OR obsessive-compulsive[Title/Abstract]) OR

disorder*[Title/Abstract]) OR hoarding[Title/Abstract]) OR panic[Title/Abstract]) OR

phobic[Title/Abstract]) OR adjustment[Title/Abstract]) OR stress[Title/Abstract])

OR posttraumatic[Title/Abstract]) OR post-traumatic[Title/Abstract]) OR "combat

disorder"[Title/Abstract]) OR generalized anxiety[Title/Abstract]))))

*And*

(((((((((((((((((((((((((((((((((((((((((((((((((((((((((((((((((((((((((((((((((((((((((((((((((((((((((((((((((((((((((((((((((((((((((((((((((((((((((((((((((Psychotherapy[MeSH Terms]) OR Drug therapy[MeSH Terms]) OR Mental health services[MeSH Terms]) OR Psychiatric somatic therapies[MeSH Terms]) OR Psychopharmacology[MeSH Terms]) OR Complementary therapies[MeSH Terms]) OR Perinatal care[MeSH Terms]) OR Maternal health services[MeSH Terms]) OR Psychotropic drugs[MeSH Terms]) OR Neurotransmitter uptake inhibitors[MeSH Terms]) OR Monoamine oxidase inhibitors[MeSH Terms]) OR Benzodiazepines[MeSH Terms]) OR

Dibenzazepines[MeSH Terms]) OR Amoxapine[MeSH Terms]) OR Amitriptyline[MeSH Terms]) OR Bupropion[MeSH Terms]) OR Buspirone[MeSH

Terms]) OR Citalopram[MeSH Terms]) OR Chlormezanone[MeSH Terms]) OR

Clomipramine[MeSH Terms]) OR Clorgyline[MeSH Terms]) OR Desipramine[MeSH Terms]) OR Desvenlafaxine succinate[MeSH Terms]) OR Dothiepin[MeSH Terms]) OR Doxepin[MeSH Terms]) OR fluoxetine[MeSH Terms]) OR Flunitrazepam[MeSH Terms]) OR Fluvoxamine[MeSH Terms]) OR Iprindole[MeSH Terms]) OR Iproniazid[MeSH Terms]) OR isocarboxazid[MeSH Terms]) OR maprotiline[MeSH Terms]) OR meprobamate[MeSH Terms]) OR moclobemide[MeSH Terms]) OR nialamide[MeSH Terms]) OR nomifensine[MeSH Terms]) OR norfenfluramine[MeSH Terms]) OR nortriptyline[MeSH Terms]) OR pargyline[MeSH Terms]) OR paroxetine[MeSH Terms]) OR phenelzine[MeSH Terms]) OR protriptyline[MeSH Terms]) OR rolipram[MeSH Terms]) OR selegiline[MeSH Terms]) OR sertraline[MeSH Terms]) OR tranylcypromine[MeSH Terms]) OR trazodone[MeSH Terms]) OR tryptophan[MeSH Terms]) OR venlafaxine hydrochloride[MeSH Terms]) OR viloxazine[MeSH Terms]) OR vilazodone[MeSH Terms]) OR Treat*[Title/Abstract]) OR Therapy[Title/Abstract]) OR Therapies[Title/Abstract]) OR Therapeu*[Title/Abstract]) OR psychotherap*[Title/Abstract]) OR Intervention*[Title/Abstract]) OR prevention[Title/Abstract]) OR preventive[Title/Abstract]) OR Support*[Title/Abstract]) OR care[Title/Abstract]) OR Drug therap*[Title/Abstract]) OR pharmacotherap*[Title/Abstract]) OR pharmacolog*[Title/Abstract]) OR Medication*[Title/Abstract]) OR psychotropic*[Title/Abstract]) OR Anti depress*[Title/Abstract]) OR anti-depress*[Title/Abstract]) OR Agomelatine[Title/Abstract]) OR Alaproclate[Title/Abstract]) OR Alprazolam[Title/Abstract]) OR Amfebutamone[Title/Abstract]) OR Amoxapine[Title/Abstract]) OR Amitriptylin*[Title/Abstract]) OR Benzodiazepin*[Title/Abstract]) OR Brofaromine[Title/Abstract]) OR Bromazepam[Title/Abstract]) OR bupropion[Title/Abstract]) OR buspiron*[Title/Abstract]) OR citalopram[Title/Abstract]) OR chlorimipramin*[Title/Abstract]) OR Chlormezanone[Title/Abstract]) OR Clomipramin*[Title/Abstract]) OR Clorazepate[Title/Abstract]) OR Clorgyline[Title/Abstract]) OR deprenyl[Title/Abstract]) OR desipramin*[Title/Abstract]) OR desvenlafaxine[Title/Abstract]) OR diazepam[Title/Abstract]) OR Dibenzazepin*[Title/Abstract]) OR Dopamine reuptake[Title/Abstract]) OR Dopamine uptake[Title/Abstract]) OR dosulepin[Title/Abstract]) OR dothiepin[Title/Abstract]) OR doxepin[Title/Abstract]) OR duloxetine[Title/Abstract]) OR escitalopram[Title/Abstract]) OR femoxetine[Title/Abstract]) OR fluoxetine[Title/Abstract]) OR flunitrazepam[Title/Abstract]) OR fluvoxamine[Title/Abstract]) OR

imipramin*[Title/Abstract]) OR iprindole[Title/Abstract]) OR

iproniazid*[Title/Abstract]) OR ipsapirone[Title/Abstract]) OR

isocarboxazid*[Title/Abstract]) OR levomilnacipran[Title/Abstract]) OR

lofepramin*[Title/Abstract]) OR lorazepam[Title/Abstract]) OR

loprazolam[Title/Abstract]) OR MAO*[Title/Abstract]) OR

maprotiline[Title/Abstract]) OR medazepam[Title/Abstract]) OR

meprobamate[Title/Abstract]) OR mianserin[Title/Abstract]) OR

milnacipran[Title/Abstract]) OR minaprine[Title/Abstract]) OR

mirtazapine[Title/Abstract]) OR moclobemide[Title/Abstract]) OR Monoamine

oxidase inhibitor*[Title/Abstract]) OR nefazodone[Title/Abstract]) OR

nialamide[Title/Abstract]) OR nitrazepam[Title/Abstract]) OR

nomifensine[Title/Abstract]) OR nordazepam[Title/Abstract]) OR Norepinephrine

reuptake[Title/Abstract]) OR Norepinephirne uptake[Title/Abstract]) OR

norfenfluramine[Title/Abstract]) OR nortriptylin*[Title/Abstract]) OR

opipramol[Title/Abstract]) OR oxazepam[Title/Abstract]) OR

paroxetine[Title/Abstract]) OR pertofrane[Title/Abstract]) OR

phenelzine[Title/Abstract]) OR pheniprazine[Title/Abstract]) OR

pirlindole[Title/Abstract]) OR pizotyline[Title/Abstract]) OR

prazepam[Title/Abstract]) OR reboxetine[Title/Abstract]) OR

rolipram[Title/Abstract]) OR selegiline[Title/Abstract]) OR Serotonin

reuptake[Title/Abstract]) OR Serotonin uptake[Title/Abstract]) OR

sertraline[Title/Abstract]) OR SNRI*[Title/Abstract]) OR SSRI*[Title/Abstract]) OR

Tetracyclic*[Title/Abstract]) OR tianeptin*[Title/Abstract]) OR

tranylcypromin*[Title/Abstract]) OR trazodone[Title/Abstract]) OR

Tricyclic*[Title/Abstract]) OR trimipramine[Title/Abstract]) OR

tryptophan[Title/Abstract]) OR venlafaxine[Title/Abstract]) OR

viloxazine[Title/Abstract]) OR vilazodone[Title/Abstract]) OR

vortioxetine[Title/Abstract]) OR zimeldine[Title/Abstract])))))))

*And*

((((((((((((infant[MeSH Terms]) OR child[MeSH Terms]) OR adolescent[MeSH Terms]) OR adult child[MeSH Terms]) OR offspring) OR baby*) OR babie*) OR newborn*) OR infant*) OR toddler*) OR child*) OR adolescent*))

*And*

((((clinical[Title/Abstract] AND trial[Title/Abstract]) OR clinical trials as topic[MeSH Terms] OR clinical trial[Publication Type] OR random*[Title/Abstract] OR random allocation[MeSH Terms] OR therapeutic use[MeSH Subheading])))

**Database: PsycINFO**

pregnancy/ or *adolescent pregnancy/ or *perinatal period/ or *postnatal period/ or *primipara/ or (pregnancy or pregnancies or pregnant or perinatal or peripartum or prenatal or prepartum or antenatal or antepartum or postnatal or postpartum or post-partum or trimester).ab,ti.

*And*

mental disorders/ or *adjustment disorders/ or *affective disorders/ or *anxiety disorders/ or *hoarding disorder/ or (depress* or mood or dysthym* or seasonal affective or anxiety or anxious or agoraphobi* or obsessive-compulsive or disorder* or hoarding or panic or phobic or adjustment or stress or posttraumatic or post-traumatic or combat disorder or generalized anxiety).ab,ti.

*And*

Treatment/ or * mental health services/ or *psychoparmacology/ or * prenatal care/ or drugs/ or antidepressant drugs/ or benzodiazepines/ or neurotransmitter uptake inhibitors/ or (treat* or therapy or therapies or therapeu* or psychotherap* or intervention* or prevention or preventive or support* or care or drug therap* or pharmacotherap* or pharmacolog* or medication* or psychotropic* or anti depress* or anti-depress* or Agomelatine or Alaproclate or Alprazolam or Amfebutamone or Amoxapine or Amitriptylin* or Benzodiazepin* or Brofaromine or Bromazepam or bupropion or buspiron* or citalopram or chlorimipramin* or Chlormezanone or Clomipramin* or Clorazepate or Clorgyline or deprenyl or desipramin* or desvenlafaxine or diazepam or Dibenzazepin* or Dopamine reuptake or Dopamine uptake or dosulepin or dothiepin or doxepin or duloxetine or escitalopram or femoxetine or fluoxetine or flunitrazepam or fluvoxamine orimipramin* or iprindole or iproniazid* or ipsapirone or isocarboxazid* or levomilnacipran or lofepramin* or lorazepam or loprazolam or MAO* or maprotiline or medazepam or meprobamate or mianserin or milnacipran or minaprine or mirtazapine or moclobemide or Monoamine oxidase inhibitor* or nefazodone or nialamide or nitrazepam or nomifensine or nordazepam or Norepinephrine reuptake or Norepinephirne uptake or norfenfluramine or nortriptylin* or opipramol or oxazepam or paroxetine or pertofrane or phenelzine or pheniprazine or pirlindole or pizotyline or prazepam or reboxetine or rolipram or selegiline or Serotonin reuptake or Serotonin uptake or sertraline or SNRI* or SSRI* or Tetracyclic* or tianeptin* or tranylcypromin* or trazodone or Tricyclic* or trimipramine or Tryptophan or venlafaxine or Viloxazine or vilazodone or Vortioxetine or zimeldine).ab,ti.

*And*

offspring/ or (offspring or baby* or babie* or newborn* or infant* or toddler* or child* or adolescent*).ab,ti.

*And*

Clinical trials/ or (clinical trial or random*).ab,ti. or clinical trial.pt.

**Database: Cochrane library**

pregnancy or pregnancies or pregnant or perinatal or peripartum or prenatal or prepartum or antenatal or antepartum or postnatal or postpartum or post-partum or trimester

*And*

depress* or mood or dysthym* or seasonal affective or anxiety or anxious or agoraphobi* or obsessive-compulsive or disorder* or hoarding or panic or phobic or adjustment or stress or posttraumatic or post-traumatic or combat disorder or generalized anxiety

*And*

treat* or therapy or therapies or therapeu* or psychotherap* or intervention* or prevention or preventive or support* or care or drug therap* or pharmacotherap* or pharmacolog* or medication* or psychotropic* or anti depress* or anti-depress* or Agomelatine or Alaproclate or Alprazolam or Amfebutamone or Amoxapine or Amitriptylin* or Benzodiazepin* or Brofaromine or Bromazepam or bupropion or buspiron* or citalopram or chlorimipramin* or Chlormezanone or Clomipramin* or Clorazepate or Clorgyline or deprenyl or desipramin* or desvenlafaxine or diazepam or Dibenzazepin* or Dopamine reuptake or Dopamine uptake or dosulepin or dothiepin or doxepin or duloxetine or escitalopram or femoxetine or fluoxetine or flunitrazepam or fluvoxamine orimipramin* or iprindole or iproniazid* or ipsapirone or isocarboxazid* or levomilnacipran or lofepramin* or lorazepam or loprazolam or MAO* or maprotiline or medazepam or meprobamate or mianserin or milnacipran or minaprine or mirtazapine or moclobemide or Monoamine oxidase inhibitor* or nefazodone or nialamide or nitrazepam or nomifensine or nordazepam or Norepinephrine reuptake or Norepinephirne uptake or norfenfluramine or nortriptylin* or opipramol or oxazepam or paroxetine or pertofrane or phenelzine or pheniprazine or pirlindole or pizotyline or prazepam or reboxetine or rolipram or selegiline or Serotonin reuptake or Serotonin uptake or sertraline or SNRI* or SSRI* or Tetracyclic* or tianeptin* or tranylcypromin* or trazodone or Tricyclic* or trimipramine or Tryptophan or venlafaxine or Viloxazine or vilazodone or Vortioxetine or zimeldine

*And*

offspring or baby* or babie* or newborn* or infant* or toddler* or child* or adolescent*

*And*

clinical trial or random*

**Database: EMBASE**

'pregnancy'/exp OR 'pregnancy' OR 'perinatal period'/exp OR 'perinatal period' OR 'prenatal period'/exp OR 'prenatal period' OR pregnancy:ab,ti OR pregnancies:ab,ti OR pregnant:ab,ti OR perinatal:ab,ti OR peripartum:ab,ti OR prenatal:ab,ti OR prepartum:ab,ti OR antenatal:ab,ti OR antepartum:ab,ti OR postnatal:ab,ti OR postpartum:ab,ti OR 'post partum':ab,ti OR trimester:ab,ti

*And*

'anxiety disorder'/exp OR 'anxiety disorder' OR 'mood disorder'/exp OR 'mood disorder' OR 'psychotrauma'/exp OR 'psychotrauma' OR depress*:ab,ti OR mood:ab,ti OR dysthym*:ab,ti OR 'seasonal affective':ab,ti OR anxiety:ab,ti OR anxious:ab,ti OR agoraphobia:ab,ti OR 'obsessive compulsive':ab,ti OR disorder*:ab,ti OR hoarding:ab,ti OR panic:ab,ti OR phobic:ab,ti OR adjustment:ab,ti OR stress:ab,ti OR posttraumatic:ab,ti OR 'post traumatic':ab,ti OR 'combat disorder':ab,ti OR 'generalized anxiety':ab,ti

*And*

‘psychiatric treatment'/exp OR 'mental health care'/exp OR 'perinatal care'/exp OR 'psychotropic agent'/exp OR treat*:ab,ti OR therapy:ab,ti OR therapies:ab,ti OR therapeu*:ab,ti OR psychotherap*:ab,ti OR intervention*:ab,ti OR prevention:ab,ti OR preventive:ab,ti OR support*:ab,ti OR care:ab,ti OR 'drug therap*':ab,ti OR pharmacotherap*:ab,ti OR pharmacolog*:ab,ti OR medication*:ab,ti OR psychotropic*:ab,ti OR 'anti depress*’:ab,ti OR 'anti depress*':ab,ti OR agomelatine:ab,ti OR alaproclate:ab,ti OR alprazolam:ab,ti OR amfebutamone:ab,ti OR amoxapine:ab,ti OR amitriptylin*:ab,ti OR benzodiazepin*:ab,ti OR brofaromine:ab,ti OR bromazepam:ab,ti OR bupropion:ab,ti OR buspiron*:ab,ti OR citalopram:ab,ti OR chlorimipramin*:ab,ti OR chlormezanone:ab,ti OR clomipramin*:ab,ti OR clorazepate:ab,ti OR clorgyline:ab,ti OR deprenyl:ab,ti OR desipramin*:ab,ti OR desvenlafaxine:ab,ti OR diazepam:ab,ti OR dibenzazepin*:ab,ti OR 'dopamine reuptake':ab,ti OR 'dopamine uptake':ab,ti OR dosulepin:ab,ti OR dothiepin:ab,ti OR doxepin:ab,ti OR duloxetine:ab,ti OR escitalopram:ab,ti OR femoxetine:ab,ti OR fluoxetine:ab,ti OR flunitrazepam:ab,ti OR fluvoxamine:ab,ti OR imipramin*:ab,ti OR iprindole:ab,ti OR iproniazid*:ab,ti OR ipsapirone:ab,ti OR isocarboxazid*:ab,ti OR levomilnacipran:ab,ti OR lofepramin*:ab,ti OR lorazepam:ab,ti OR loprazolam:ab,ti OR mao*:ab,ti OR maprotiline:ab,ti OR medazepam:ab,ti OR meprobamate:ab,ti OR mianserin:ab,ti OR milnacipran:ab,ti OR minaprine:ab,ti OR mirtazapine:ab,ti OR moclobemide:ab,ti OR 'monoamine oxidase inhibitor*':ab,ti OR nefazodone:ab,ti OR nialamide:ab,ti OR nitrazepam:ab,ti OR nomifensine:ab,ti OR nordazepam:ab,ti OR 'norepinephrine reuptake':ab,ti OR 'norepinephirne uptake':ab,ti OR norfenfluramine:ab,ti OR nortriptylin*:ab,ti OR opipramol:ab,ti OR oxazepam:ab,ti OR paroxetine:ab,ti OR pertofrane:ab,ti OR phenelzine:ab,ti OR pheniprazine:ab,ti OR pirlindole:ab,ti OR pizotyline:ab,ti OR orprazepam:ab,ti OR reboxetine:ab,ti OR rolipram:ab,ti OR selegiline:ab,ti OR 'serotonin reuptake':ab,ti OR 'serotonin uptake':ab,ti OR sertraline:ab,ti OR snri*:ab,ti OR ssri*:ab,ti OR tetracyclic*:ab,ti OR tianeptin*:ab,ti OR tranylcypromin*:ab,ti OR trazodone:ab,ti OR tricyclic*:ab,ti OR trimipramine:ab,ti OR tryptophan:ab,ti OR venlafaxine:ab,ti OR viloxazine:ab,ti OR vilazodone:ab,ti OR vortioxetine:ab,ti OR zimeldine:ab,ti

*And*

'child'/exp OR 'adolescent'/exp OR 'newborn period'/exp OR 'childhood'/exp OR 'adolescence'/exp OR 'adult child'/exp OR 'progeny'/exp OR offspring:ab,ti OR baby*:ab,ti OR babie*:ab,ti OR newborn*:ab,ti OR infant*:ab,ti OR toddler*:ab,ti OR child*:ab,ti OR adolescent*:ab,ti

*And*

random*:ab,ti OR 'clinical trial':it,lnk,ab,ti OR exp AND ('health'/exp OR health)
